# Nematicidal actions of the marigold exudate α-terthienyl: oxidative stress-inducing compound penetrates nematode hypodermis

**DOI:** 10.1242/bio.038646

**Published:** 2019-03-29

**Authors:** Takahiro Hamaguchi, Kazuki Sato, Cláudia S. L. Vicente, Koichi Hasegawa

**Affiliations:** 1 Department of Environmental Biology, College of Bioscience & Biotechnology, Chubu University, 1200 Matsumoto, Kasugai, Aichi 487-8501, Japan; 2 RIKEN Center for Sustainable Resource Science, 1-7-22 Suehiro-cho, Tsurumi-ku, Yokohama, Kanagawa 230-0045, Japan; 3 NemaLab/ICAAM - Instituto de Ciências Agrárias e Ambientais Mediterrânicas, Departamento de Biologia, Universidade de Évora, Núcleo da Mitra, Ap. 94, 7002-554 Évora, Portugal

**Keywords:** Allelochemical, *Caenorhabditis elegans*, Glutathione S-transferase, Superoxide dismutase, SKN-1, WDR-23

## Abstract

α-terthienyl is an allelochemical derived from the roots of marigold (*Tagetes* spp.), which is used to suppress plant parasitic nematodes. We investigated the nematicidal activity of α-terthienyl against the model organism *Caenorhabditis elegans* and the root-knot nematode, *Meloidogyne incognita.* As reported previously, α-terthienyl action was much higher after photoactivation, but was still effective against *C. elegans* dauer larvae and *M. incognita* second stage juveniles, even without photoactivation. Expression induction of two major enzymes, glutathione S-transferase (GST) and superoxide dismutase (SOD), was restricted in *C. elegans* hypodermis following treatment with α-terthienyl. The susceptibility of nematodes to α-terthienyl changed when the expression of GST and SOD was induced or suppressed. From these results, under dark conditions (without photoactivation), α-terthienyl is an oxidative stress-inducing chemical that effectively penetrates the nematode hypodermis and exerts nematicidal activity, suggesting high potential for its use as a practicable nematode control agent in agriculture.

## Abstract (precis)

**Summary:** α-terthienyl, an exudate of the marigold, effectively penetrates the nematode hypodermis in *Caenorhabditis elegans* dauer and *Meloidogyne incognita* J2 larvae, and exerts nematicidal activity through oxidative stress induction.

## INTRODUCTION

Global agricultural losses caused by plant parasitic nematodes (PPNs) have been estimated at US$100 billion annually (Kushida and Kondo, 2015; Oka et al., 2000). As the availability of registered nematicides are being sequentially limited, development of alternative management strategies against PPNs is urgently required, with an emphasis on eco-friendly options (Wang et al., 2007). The concept of integrated pest management (IPM), which combines both conventional and new control strategies, has become important (Oka et al., 2000). Crop rotations, such as cotton-peanut, are well established and widely used for nematode management (Jordan et al., 2008). Since some varieties of marigolds (*Tagetes* spp.) are reported to be resistant against root-knot nematodes (RKN), scientists have been studying their allelopathic potential toward PPNs and applications as an antagonistic plant (Steiner, 1941; Hooks et al., 2010). While marigolds have utility as antagonistic plants, their nematicidal action is not well understood when incorporated into the soil as crop residue or root extract (Wang et al., 2007). In addition, their usefulness as antagonistic plants is still largely restricted because marigolds are strongly affected by changes in temperature and day length (Wang et al., 2007).

A blue-fluorescing compound, α-terthienyl (C_12_H_8_S_3_, MW 248.376), was isolated from marigolds and characterized (Zechmeister and Sease, 1947). Later, α-terthienyl was recognized for its nematicidal, insecticidal, fungicidal, antiviral and cytotoxic activities, and is now believed to be the main compound responsible for the nematicidal activity of marigold (Wang et al., 2007). α-terthienyl is believed to be a potential candidate for non-residual and environmentally friendly pesticides (Wang et al., 2007; Nivsarkar et al., 2001), however, additional research is required.

Several mechanisms of α-terthienyl activity have been elucidated. The oxygen-dependent phototoxicity of α-terthienyl reportedly generates singlet oxygen and superoxide anion radicals (Nivsarkar et al., 2001). These reactive oxygen species (ROS) *in vivo* are substrates for the detoxification enzymes superoxide dismutases (SODs) and catalases (CTLs). In addition, photoactivated α-terthienyl (PAT) showed significant concentration-dependent ROS-induction activity in lepidopteran ovarian Tn5B1-4 and Sf-21 cells, which decreased the activity of peroxidase (POD), SOD and CTL (Huang et al., 2017). Since biomolecules oxidized by ROS also become harmful substances, phase II metabolism that employs such enzymes as glutathione S-transferases (GSTs) and UDP-glucuronosyl transferases (UGTs) (Lindblom and Dodd, 2006) is thought to be important for resistance to α-terthienyl. Oxidative stress-related enzymes in the free-living model nematode *Caenorhabditis elegans* are largely controlled by the cap‘n’collar transcription factor SKN-1, which is a structural and functional homolog of the mammalian Nef2 (An and Blackwell, 2003). SKN-1 activity in such stress responses is negatively regulated by the WD40 repeat protein, WDR-23 (Choe et al., 2009).

Although α-terthienyl is reportedly a photoactivated chemical (Nivsarkar et al., 2001), nematicidal activity occurs even without photoactivation (Faizi et al., 2011). If α-terthienyl is to be incorporated into agricultural fields as a nematicidal agent, photoactivation is unlikely to occur. In this study, we used *C. elegan*s as a model organism for PPN research (Jones et al., 2011; Gillet et al., 2017) to understand the nematicidal action of α-terthienyl with and without photoactivation. Here we show that α-terthienyl (1) has nematicidal activity even without photoactivation, (2) is more effective against dauer larvae than adults, (3) penetrates the nematode hypodermis and exerts its action and (4) acts through oxidative stress.

## RESULTS

### Nematicidal activity of α-terthienyl with or without photoactivation

**Fig. 1.**
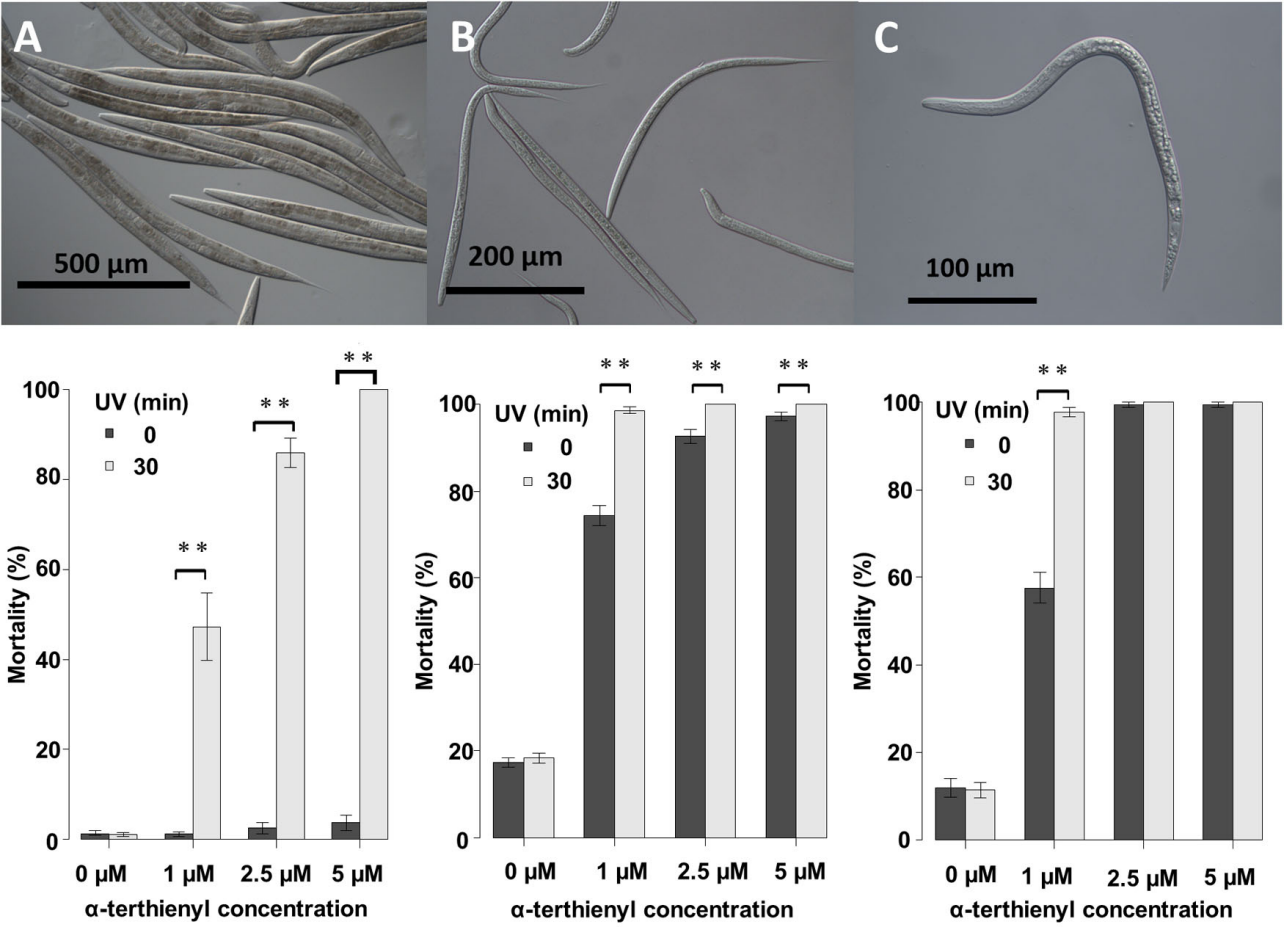
**Mortality of nematodes after exposure to different α-terthienyl concentrations with or without photoactivation.** Mortality percentages (mean±s.e.) of *C. elegans* young adults (A), *C. elegans* dauer larvae (B), and *M. incognita* J2s (C), after exposure to different α-terthienyl concentrations, with or without 30 min photoactivation. Mortality was counted after 24 h. (**P<0.005, Mann–Whitney *U*-test). Mean±s.e. of each mortality percentage was calculated from the totals of over 12 wells from 96-well experiments; more than four wells were used in one experiment (total >80 nematodes per sample) and these were repeated three times independently. Photos were taken using DIC microscopy.

We first examined the nematicidal activity of α-terthienyl against *C. elegans* young adults and dauer larvae, and second juvenile stage (J2) of *M. incognita*, with or without photoactivation. Normally these nematodes are considered to live as dauer larvae or in the J2 stage in the soil when food sources are poor. Although nematicidal activity of α-terthienyl against *C. elegans* young adults was not observed at concentrations of 0, 1, 2.5 or 5 μM after 24 h without photoactivation, its activity dramatically increased at all concentrations when photoactivated (Fig. 1A). Mortality percentages of *C. elegans* young adults were 47±7.2% at 1 μM, 86±3.1% at 2.5 μM, 100±0.0% at 5 μM (mean±s.e.), and lethal concentration 50 (LC_50_) was 1.93±0.03 μM. The dauer larval stage is a non-feeding stage, entirely covered with a tough cuticle that confers resistance against environmental stresses, including harmful chemicals (Cassada and Russell, 1975). The second juvenile stage (J2) of *M. incognita* is a quiescent and infective stage found freely in the soil (Karssen and Moens, 2006). Surprisingly *C. elegans* dauer larvae and *M. incognita* J2s were effectively killed by α-terthienyl treatment even without photoactivation, although its activity increased when photoactivated (Fig. 1B,C). When treated with α-terthienyl without photoactivation, the mortality of *C. elegans* dauer larvae was 74±2.2% at 1 μM, 93±1.5% at 2.5 μM, 97±0.88% at 5 μM, and LC_50_ was 0.72±0.06 μM. When treated with α-terthienyl after photoactivation, mortality of *C. elegans* dauer larvae was 99±0.76% at 1 μM, 100±0.0% at 2.5 μM, 100±0.0% at 5 μM, and LC_50_ was 0.28±0.02 μM. When treated with α-terthienyl without photoactivation, mortality of *M. incognita* J2s was 58±3.3% at 1 μM, 99±0.55% at 2.5 μM, 99±0.55% at 5 μM, and LC_50_ was 0.84±0.05 μM. When treated with α-terthienyl after photoactivation, mortality of *M. incognita* J2s was 98±1.0% at 1 μM, 100±0.0% at 2.5 μM, 100±0.0% at 5 μM, and LC_50_ was 0.37±0.03 μM.

### Nematicidal activity of non-photoactivated α-terthienyl against *C. elegans* adult and dauer larvae

**Fig. 2.**
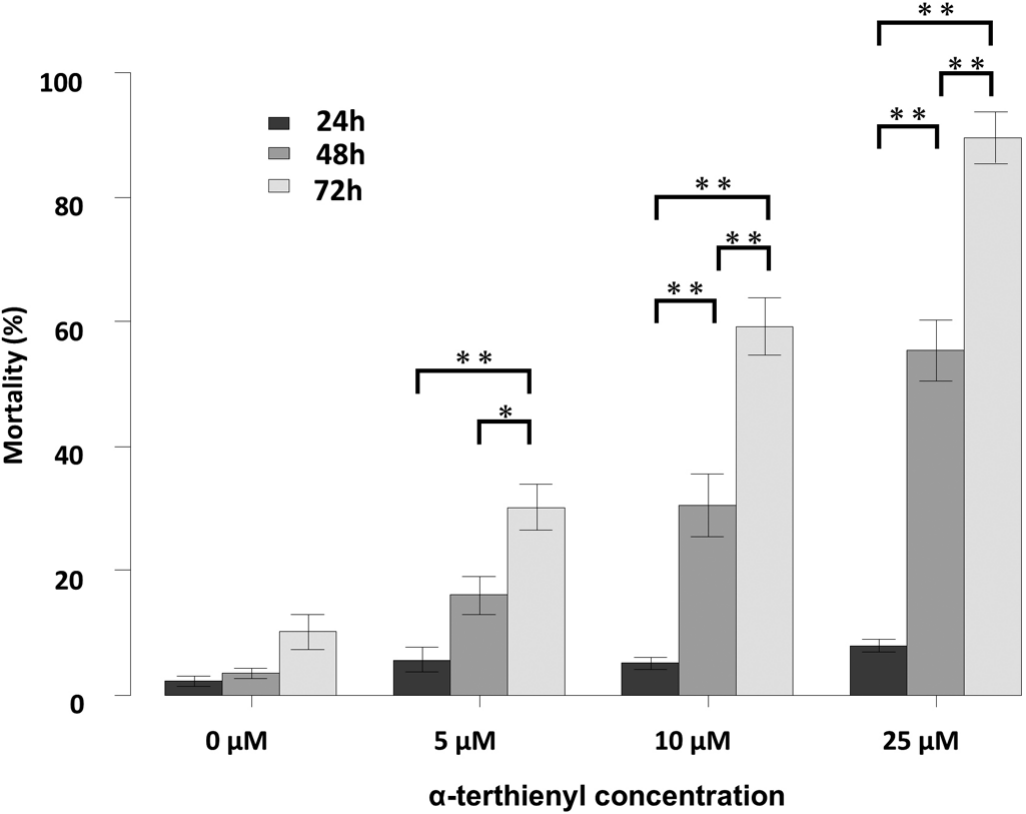
**Mortality of *C. elegans* after exposure to different α-terthienyl concentrations without photoactivation.** Mortality percentages (mean± s.e.) of *C. elegans* young adults, 24, 48 and 72 h after α-terthienyl exposure. Mean±s.e. of each mortality percentage was calculated from over 12 wells from 96-well experiments; more than four wells were used in one experiment (total >80 nematodes per sample) and these were repeated three times independently (*P<0.05, **P<0.005, Mann–Whitney *U*-test followed by Bonferroni correction).

We treated *C. elegans* young adults with α-terthienyl for 24, 48 and 72 h without photoactivation. Nematicidal activity of α-terthienyl was not observed at any concentration after 24 h, but mortality increased notably after 48 and 72 h in a dose-dependent manner (Fig. 2). After 48 h, mortality of nematodes was 16±3.0% at 5 μM, 31±4.8% at 10 μM, 55±4.8% at 25 μM, and LC_50_ was 22±1.1 μM. After 72 h, mortality was 30±3.5% at 5 μM, 59±4.4% at 10 μM, 90±4.1% at 25 μM, and LC_50_ was 11±0.52 μM.

### Expression patterns of detoxification enzymes against α-terthienyl

The major enzyme families against ROS are SODs, glutathione peroxidases (GPXs), CTLs and peroxiredoxins (PRDXs) (Henkle-Dührsen and Kampkötter, 2001). In addition, biological molecules oxidized by ROS also became harmful substances and were detoxified by phase II metabolism employing such enzymes as GSTs and UGTs (Lindblom and Dodd, 2006). To study the molecular responses of *C. elegans* to α-terthienyl, we selected SOD-1, CTL-1 and GST-4 as representatives of the enzyme families since expression of these against oxidants and xenobiotics are relatively well documented (Doonan et al., 2008; Hasegawa et al., 2008; Choe et al., 2009; Vicente et al., 2015).

**Fig. 3.**
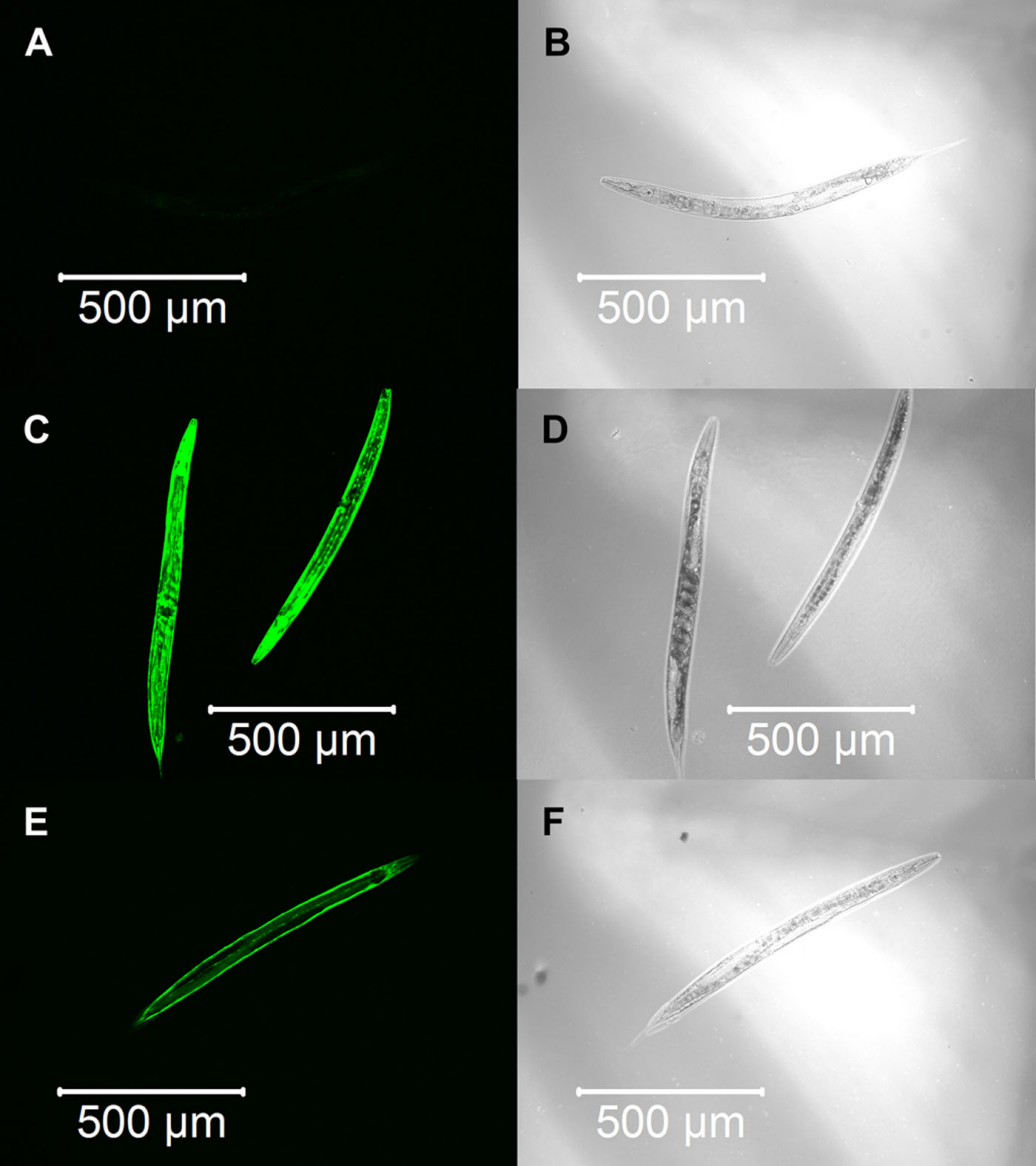
**GST-4::GFP expression patterns in the transgenic nematode KHA143 *{chuIs143[unc-119(+), Pgst-4::gst-4::gfp]II}*.** (A,B) No expression was observed without any treatment. (C,D) Strong GST-4::GFP expression was induced throughout the body when treated with 7.03 mM of acrylamide for 24 h. (E,F) GST-4::GFP expression was induced in the hypodermis when treated with 10 µM of α-terthienyl for 24 h.

**Fig. 4.**
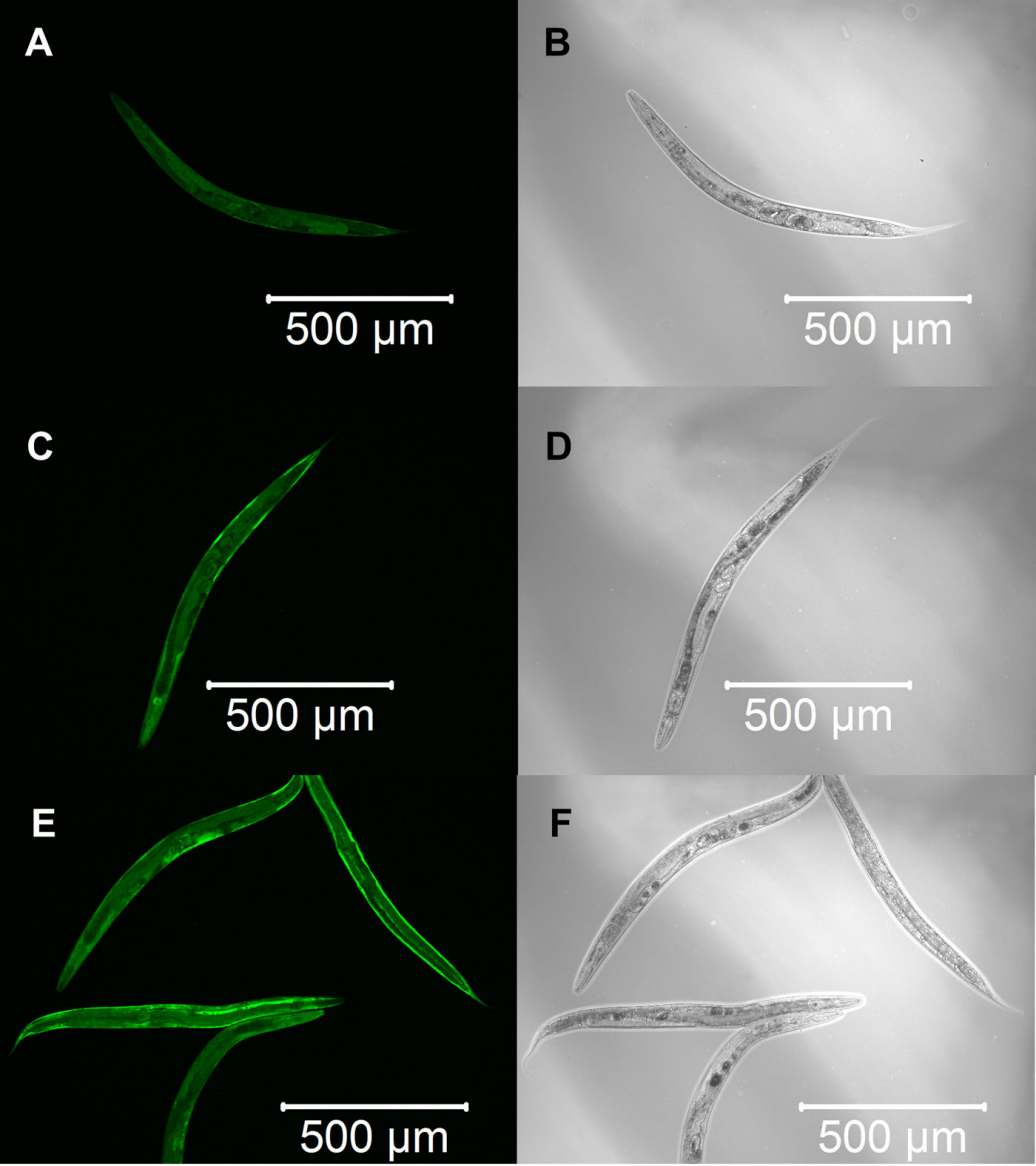
**SOD-1::GFP expression patterns in the transgenic nematode KHA169 {*sod-1::[chuSi169 gfp::3×flag]II}*.** (A,B) Weak and constitutive expression of SOD-1::GFP was observed throughout the body without any treatment. (C,D) Strong SOD-1::GFP expression was induced throughout the body when treated with 7.03 mM of acrylamide for 24 h. (E,F) SOD-1::GFP expression was induced strongly in the hypodermis when treated with 10 µM of α-terthienyl for 24 h.

**Fig. 5.**
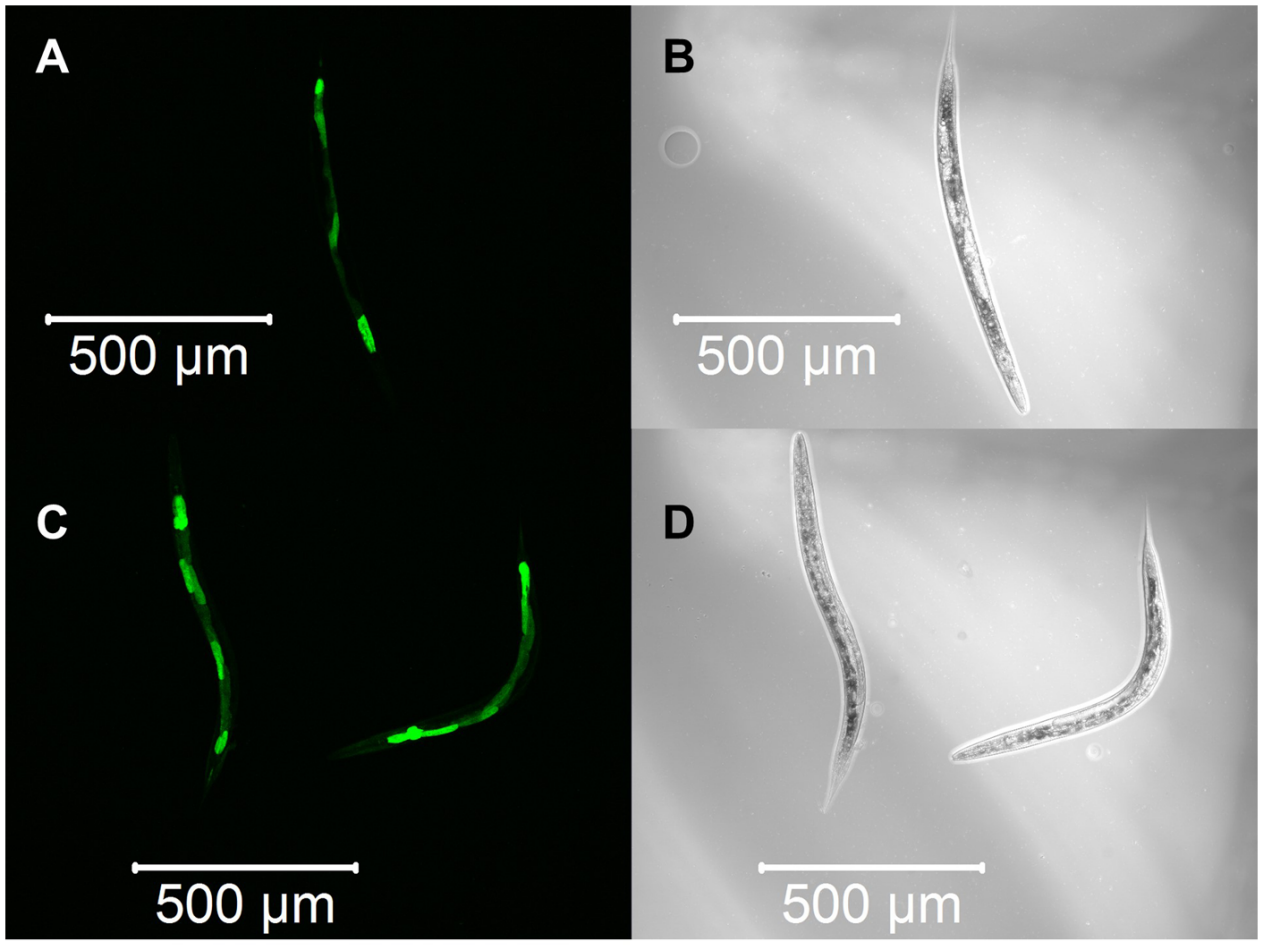
**CTL-1::GFP expression patterns in the transgenic nematode KHA166 *{chuIs166[unc-119(+), Pctl-1::Bxy-ctl-1::gfp]}*.** (A,B) Constitutive expression of CTL-1::GFP was observed in the intestine without any treatment. (C,D) Expression patterns of CTL-1::GFP were not affected when treated with 10 µM of α-terthienyl for 24 h.

We constructed three transgenic *C. elegans* expressing detoxification enzymes tagged with GFP reporter proteins, GST-4::GFP, SOD-1::GFP or CTL-1::GFP, and observed the expression patterns after α-terthienyl treatment. GST-4 was not expressed without stress (Fig. 3A,B) and its expression was induced strongly throughout the body after treatment with effective GST inducer acrylamide (Fig. 3C,D). When the *gst-4::gfp* transgenics were treated with α-terthienyl, GST-4 was strongly expressed, mainly in the hypodermis (Fig. 3E,F). Without stress, SOD-1 was constitutively but weakly expressed throughout the body (Fig. 4A,B), and was slightly induced throughout the body after treatment with acrylamide (Fig. 4C,D). SOD-1 expression was also induced, particularly in the hypodermis, when *sod-1::gfp* transgenics were treated with α-terthienyl (Fig. 4E,F). CTL-1 (catalase), was constitutively expressed in gut cells (Fig. 5A,B) but was not affected when treated with α-terthienyl (Fig. 5C,D). In addition to these expression patterns, quantitative analyses of these enzymes were also performed and the same trends were observed (Figs S1 and S2).

To analyze GST-4 expression patterns at the single-cell level, we fused *gst-4* with *nls* (nuclear localization signal) and *rfp* (red fluorescence protein) and transformed it into *C. elegans*. We confirmed again that GST-4 was not expressed under non-stress conditions (data not shown), but was strongly induced in all somatic cells after treatment with acrylamide (Fig. S3A,B); its expression was induced and restricted in hypodermal cells when treated with α-terthienyl (Fig. S3C,D).

### Expression of the detoxification enzymes against α-terthienyl under the control of the SKN-1/WDR-23 system

**Fig. 6.**
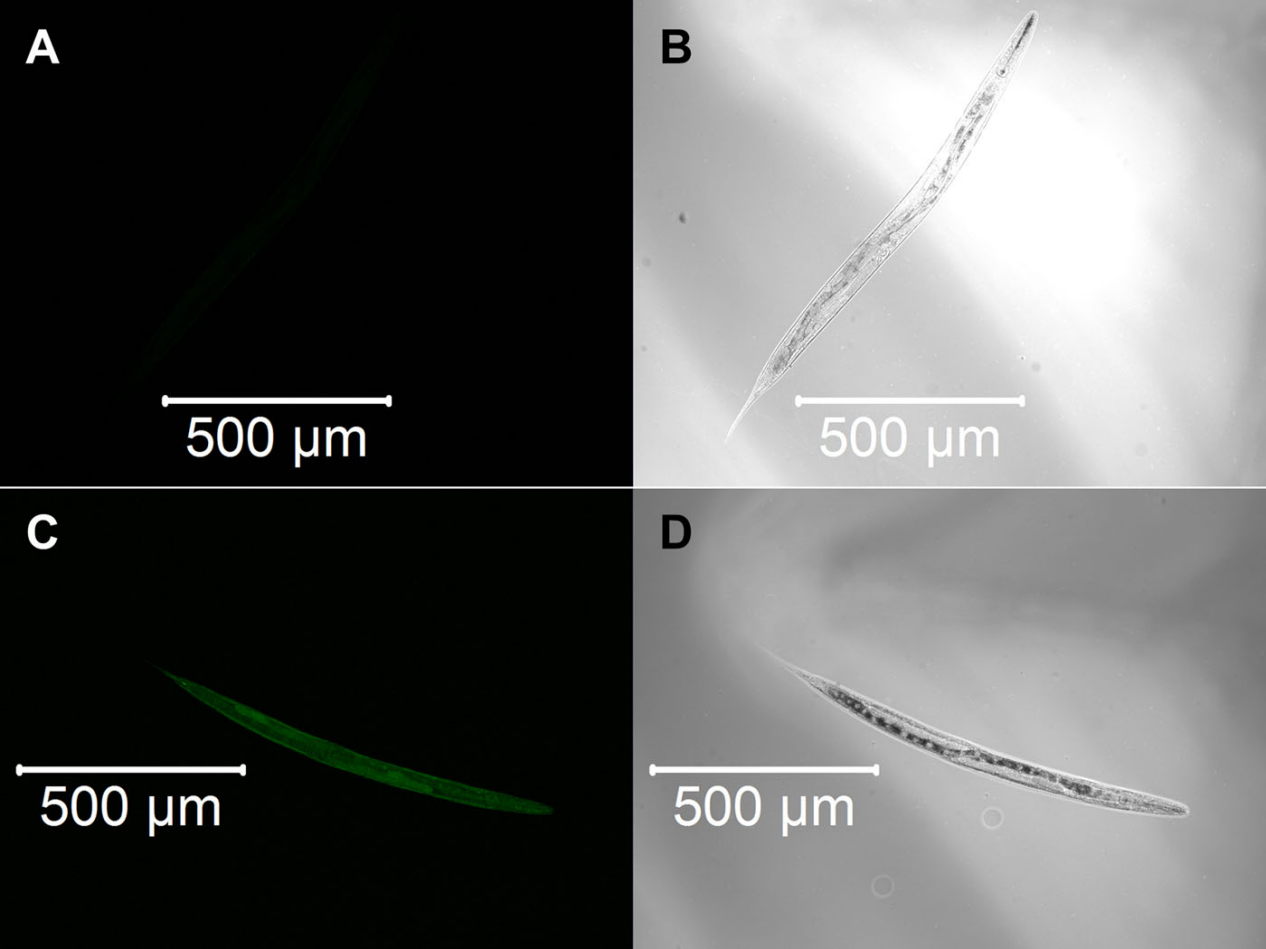
**Knockdown of *skn-1* by RNAi suppresses the induction of GST-4::GFP and SOD-1::GFP expression even after treatment with 10 µM α-terthienyl for 24 h.** (A,B) No expression of GST-4::GFP in KHA143 *{chuIs143[unc-119(+), Pgst-4::gst-4::gfp]II}* was observed even after treatment with 10 µM of α-terthienyl for 24 h. (C,D) Weak and constitutive expression of SOD-1::GFP in KHA169 *{sod-1::gfp [chuSi169 gfp::3×flag]II}* after treatment with 10 µM of α-terthienyl for 24 h.

**Fig. 7.**
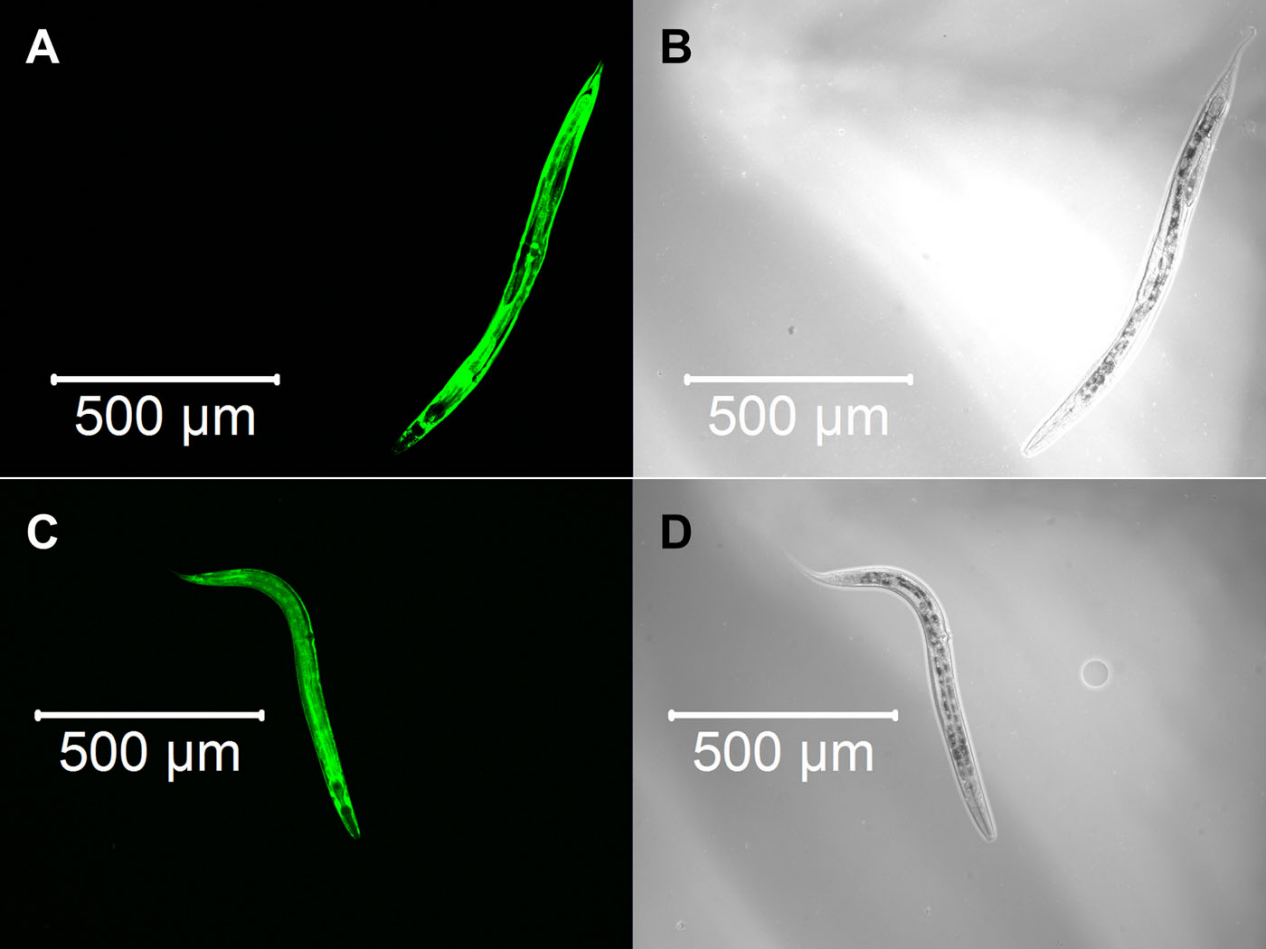
**Knockdown of *wdr-23* by RNAi strongly induced GST-4::GFP and SOD-1::GFP expressions throughout the body.** (A,B) Strong expression of GST-4::GFP in KHA143 *{chuIs143[unc-119(+), Pgst-4::gst-4::gfp]II}* was observed throughout the body. (C,D) Strong expression of SOD-1::GFP in KHA169 *{sod-1::gfp [chuSi169 gfp::3×flag]II}* was observed throughout the body.

SKN-1 is a member of the CNC (cap‘n’collar) family of transcription factors and directly controls expression of many detoxification enzymes. WD40 repeat protein WDR-23 represses/releases SKN-1 activity depending on environmental conditions (Tang and Choe, 2015). We analyzed the involvement of these two molecules in *gst-4* and *sod-1* expression in response to α-terthienyl. When *skn-1* was knocked down, inductions of GST-4 and SOD-1 in the nematode hypodermis by α-terthienyl treatment was suppressed (Fig. 6A–D). We also confirmed that *skn-1* RNAi suppressed expression of both enzymes, except for in muscles, even after treatment with acrylamide (Fig. S4A–D). When *wdr-23* was knocked down, expression of GST-4 and SOD-1 was strongly induced without chemical treatment (Fig. 7A–D) and this expression did not change after α-terthienyl treatment (data not shown). No expression changes in CTL-1 were observed when both *skn-1* and *wdr-23* were knocked down (data not shown).

### Expression of the detoxification enzymes are responsible for nematode susceptibility to α-terthienyl

**Fig. 8.**
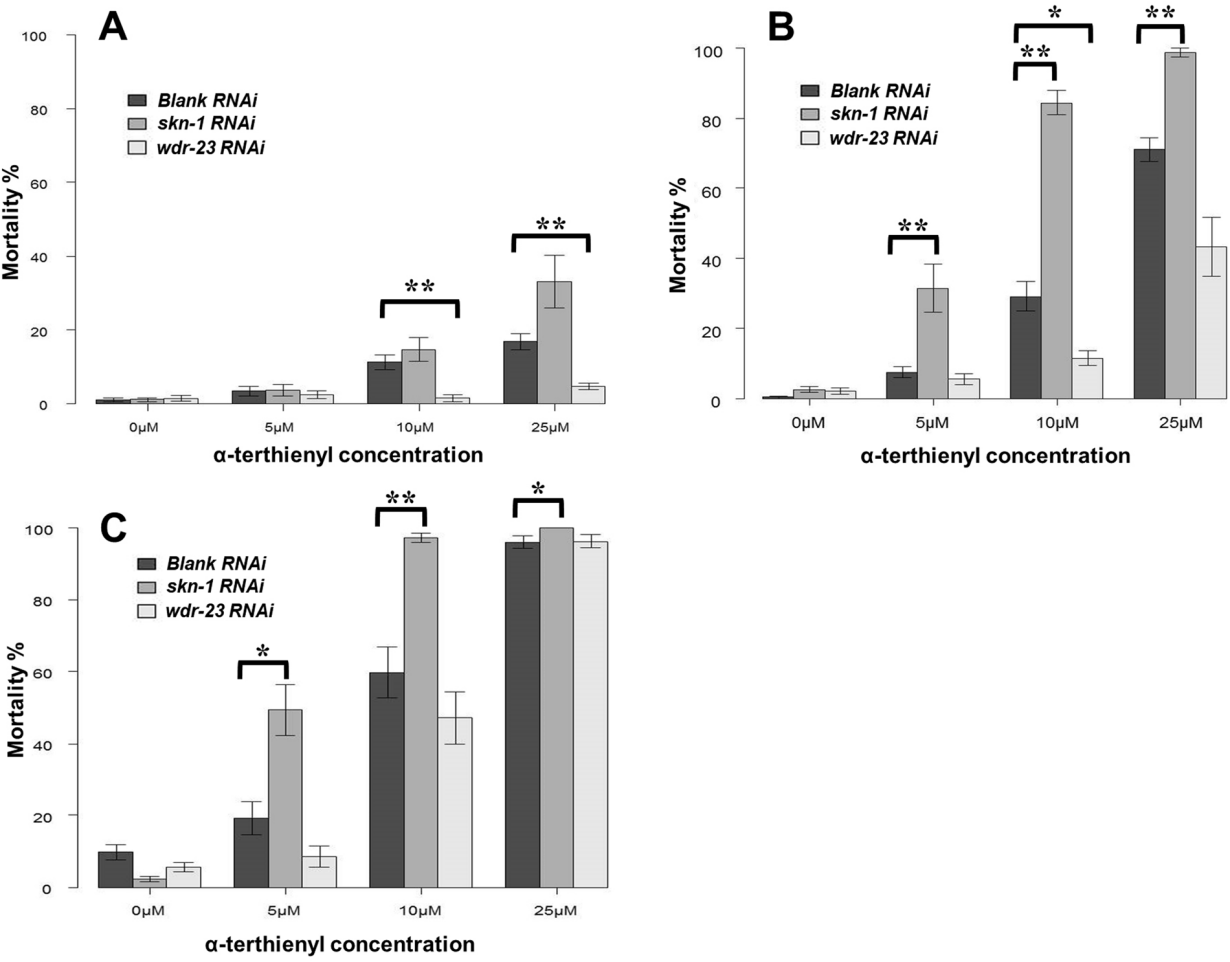
***C. elegans* susceptibility to α-terthienyl was affected by the activities of *skn-1* and *wdr-23*.** Mortality percentage (mean±s.e.) of nematodes treated by *RNAi* after exposure of α-terthienyl for (A) 24 h, (B) 48 h and (C) 72 h. Mean±s.e. of each mortality percentage was calculated from over 12 wells of 96-well experiments; more than four wells were used in one experiment (total >80 nematodes per sample) and these were repeated three times independently (*P<0.05, **P<0.005, Mann–Whitney *U*-test followed by Bonferroni correction).

We analyzed the involvement of the detoxification enzymes in *C. elegans* susceptibility to α-terthienyl using *skn-1/wdr-23* RNAi. As confirmed before, the nematicidal activity of α-terthienyl was observed to be dose- and time-dependent when treated with blank RNAi (control). When *skn-1* and *wdr-23* were knocked down, mortality of nematodes was clearly affected (Fig. 8A–C). At concentrations of 10 μM and 25 μM of α-terthienyl after 24 h, mortality of nematodes was 11±2.0% and 17±2.1%, respectively, but mortality was up to 15±3.1% and 33±6.8% if *skn-1* was knocked down, and mortality was down to 1.5±0.92% and 4.7±0.83% if *wdr-23* was knocked down (Fig. 8A). Differences in mortality were more prominent after 48 h (Fig. 8B), but almost all nematodes died when treated with 25 μM of α-terthienyl under all RNAi conditions after 72 h (Fig. 8C).

## DISCUSSION

α-terthienyl is photoactivated generator of singlet oxygen and superoxide anion radicals (Huang et al., 2017; Nivsarkar et al., 2001). Mosquito larval mortality was 100% after treatment with naturally extracted or commercially synthesized α-terthienyl at concentrations of 33 ppb (≈0.13 µM) and ultraviolet excitation (366 nm) (Nivsarkar et al., 2001). No difference was observed between light-excited and non-treated α-terthienyl against the cyst nematode *H. zeae*; mortality of *H. zeae* was 100% when treated with commercially available α-terthienyl at concentrations of 0.125% (≈5 mM) for 24 h (Faizi et al., 2011). Our experiments clearly indicated that photoactivated α-terthienyl shows much greater toxicity against *C. elegans* young adults, dauer larvae, and *M. incognita* J2s than that without photoactivation. Since α-terthienyl is expected to exert nematicidal action in the soil, we investigated in detail the action of the chemical without photoactivation.

SODs are enzymes that detoxify superoxide (O_2_^−^) radicals into oxygen (O_2_) and hydrogen peroxide (H_2_O_2_), followed by the detoxification of H_2_O_2_ to H_2_O and O_2_ by CTLs. Phase II enzymes catalyze reactions that conjugate critical reducing agents with reactive xenobiotics and/or oxidative biomolecules metabolized by Phase I enzymes (An and Blackwell, 2003). Biological defense systems against such harmful molecules are broadly conserved in animals. In mammals, NF-E2-related factor 2 (Nrf2) is a central transcription factor involved in the transcriptional activation of many genes encoding Phase II enzymes via the antioxidant response element (Nguyen et al., 2003). Functional similarity of *C. elegans* SKN-1 to mammalian Nrf has been reported (An and Blackwell, 2003). In *C. elegans*, SKN-1 is a transcription factor required for response to oxidative stresses; SKN-1 activation induces the expression of *ctl*, *sod*, and at least nine *gst* genes (Park et al., 2009). The SKN-1 binding site is present in the promoter regions of many Phase II detoxification enzymes and also that of *ctl* and *sod* (An and Blackwell, 2003; Lindblom and Dodd, 2006). Steady-state mRNA levels of the *gst-4* increased 40-fold, and the *sod-1* and *-3* increased twofold in the *C. elegans* homogeneous larval population in response to exposure to an oxidative stress inducer paraquat (Tawe et al., 1998).

Nuclear accumulation of SKN-1 is repressed by WDR-23 which is expressed in intestinal-, hypodermal- and neuronal-cell nuclei and interacts with DDB-1 and SKN-1 (Choe et al., 2009). Loss of function of WDR-23 causes constitutive transcription of Phase II detoxification genes, accumulation of SKN-1 in the intestinal nuclei, and elevation of SKN-1 protein levels (Choe et al., 2009). *skn-1* RNAi prevented GST-4 expression induced by the strong GST inducer acrylamide except for that in the pharynx and body wall muscle (Hasegawa et al., 2008). In addition, the upregulations of *sod-1* and *ctl-2* were inhibited 80% and 100%, respectively, by *skn-1* RNAi (Park et al., 2009). Since GST-4 and SOD-1 were induced in the *C. elegans* hypodermis after treatment with α-terthienyl (Figs 3 and 4), this chemical quickly permeates the nematode cuticle and acts as an oxidative stressor. If α-terthienyl is taken by mouth, expression of these detoxifying enzymes should also be observed in the pharyngeal muscle and intestine, as observed in the transgenics treated with acrylamide (Figs 4 and 5; Fig. S1). We confirmed that the susceptibility of nematodes to α-terthienyl increased when expression of GST-4 and SOD-1 was suppressed by *skn-1* RNAi. Furthermore, we found that knocking down of *wdr-23* induced GST-4 and SOD-1 expression and conferred resistance against α-terthienyl. CTL-1 is constitutively expressed in the nematode intestine, but its expression was not induced when transgenic KHA166 (*ctl-1::gfp*) was treated with α-terthienyl. We also confirmed that CTL-1 was not affected neither by the RNAi of either *skn-1* or *wdr-23* (data not shown). This result supports the hypothesis that the defense system against α-terthienyl is largely dependent on the SKN-1/WDR-23.

Furthermore, we found that dauer larvae exhibited much greater sensitivity to α-terthienyl (Figs 1 and 2). *C. elegans* dauer larvae are morphologically and metabolically specialized to be able to survive harsh conditions (Cassada and Russell, 1975; Erkut and Kurzchalia, 2015). Vanfleteren and De Vreese (1995) found that SOD and CTL activity was elevated in dauer larvae, which is in contrast to our results. Although constitutive expression of these enzymes is more frequent in the dauer stage than in the propagative stage, their inducible responses against xenobiotics might be stagnant. We did not observe induction of GST-4 and SOD-1 expression in dauer larvae of the transgenics after α-terthienyl treatment (data not shown). From these results, we concluded that α-terthienyl is an oxidative stress-inducing chemical that effectively penetrates the nematode hypodermis even in the dauer/infectious larvae and exerts nematicidal activity.

Other plants with nematicidal properties have been reported and these are believed to release nematicidal compounds when incorporated into the soil. 1,2-dehydropyrrolizidine alkaloids (PAs) present in Crotalaria are used in nematode control (Ntalli and Caboni, 2012). One of the main allelochemical compounds of monocrotaline has harmful effects on livestock and humans, damaging the central nervous system (Ntalli and Caboni, 2012). A new active compound 6′-methyl-fungichromin (fungichromin B) extracted from the potent nematicidal bacteria *Streptomyces albogriseolus* HA10002 could also be a promising candidate as a natural microorganism-based product (Zeng et al., 2013). These practicalities could also be tested on the model nematode *C. elegans*.

In this model system we show that α-terthienyl (1) has nematicidal activity even without photoactivation, (2) is more effective against dauer larvae than adults, (3) penetrates the nematode hypodermis and exerts effects, and (4) acts through oxidative stress. This substance would be effective even in soils with limited or no light, and exerts its nematicidal actions against non-feeding larvae by penetrating their cuticle, suggesting high potential for practicable use as an agricultural nematode control agent.

## MATERIALS AND METHODS

### Nematode strains and culturing

*C. elegans* culturing and handling were performed as described previously (Brenner, 1974; Stiernagle, 2006), except when noted otherwise. Briefly, nematodes were cultured on nematode growth medium (NGM), seeded with *E.* *coli* OP50 as a food source and maintained at 20°C. Strains used in this experiments were: wild-type N2 (Bristol strain); DP38 *unc-119(ed3)III*; KHA143 *{chuIs143[unc-119*(+)*, Pgst-4::gst-4::gfp]II}* (Transgenic nematode expressed *gst-4::gfp* fusion gene under the control of *gst-4* promoter); KHA166 *{chuIs166[unc-119(+), Pctl-1::Bxy-ctl-1::gfp]}* (Transgenic nematode expressed *ctl-1::gfp* fusion gene under the control of *ctl-1* promoter); KHA117 *{chuIs117[unc-119*(+)*, Pgst-4::gst-4::nls::rfp]I}* (Transgenic nematode expressed *gst-4::nls::rfp* fusion gene under the control of *gst-4* promoter), and KHA169 {*sod-1::[chuSi169 gfp::3×flag]II}* (Transgenic nematode expressed *sod-1::gfp* fusion gene under the control of *sod-1* promoter). These transgenic nematodes, except for KHA169 (*sod-1::gfp*), were generated by conventional *C. elegans* transformation techniques (Evans, 2006). The transgenic animal KHA169 (*sod-1::gfp*) was generated by gene-tagging with a self-exciting drug selection cassette (Dickinson et al., 2015). Detailed information for the transgenics are described below.

Synchronized L1 stage *C. elegans* were obtained by treating egg-containing adults with sodium hypochlorite (Porta-de-la-Riva et al., 2012). They were allowed to grow on NGM plates seeded with *E. coli* OP50 at 20°C for 48 h until they reached the young adult stage. Dauer larvae of *C. elegans* were collected from old NGM plates with M9 buffer (after culture four weeks at 20°C).

*Meloidogyne incognita* (originally isolated from Miyakonojo, Japan) were maintained on the tomato cultivar Chibikko in a hydroponic culture system (Nishiyama et al., 2015) in Ryoji Shinya laboratory (Meiji University, Japan). Hatched second stage juveniles (J2s) were collected using the Baermann funnel technique and used for experiments within a week.

### Transgenic *C. elegans* construction

To make reporter constructs, all PCRs were performed with Takara PrimeSTAR GXL DNA polymerase (Takara Bio Inc., Japan). To make the transgenic nematode expressing the *gst-4::gfp* fusion gene under the control of *gst-4* promoter, we first amplified the *gst-4* promoter and CDS (coding sequence) using PCR with the primers, GST4For_SalI, 5′-GGG TCG ACT TTT GCA GAC TAA AAA TAA CTA CTC TG-3′ and GST-4Rev_BamH1, 5′-GGG GAT CCA ACA ATA CTA TCC TTT CTT GTT GCC-3′ from the genomic DNA of the *C. elegans* N2. Amplicons were ligated into the pPD95.77 *gfp* expression vector (kindly provided by A. Fire, Stanford University) to obtain the *Pgst-4::gst-4::gfp* construct. The construct (100 µg/ml) was co-injected into the gonadal arms of *unc-119(ed3)* adult hermaphrodites, with an equal concentration of pDP#MM016B, to obtain transgenics, having transgenes as an extrachromosomal array. After integration of extrachromosomal arrays into chromosomes (Mitani, 1995), animals were outcrossed three times with N2 to obtain KHA143 *{chuIs143[unc-119(+), Pgst-4::gst-4::gfp]II}*. We mapped the integrated transgene to LG II using conventional genetic markers *dpy-5(e61)I*, *unc-4(e120)II*, *dpy-18(e364)III*, *unc-24(e138) IV*, *unc-42(e270)V*, *lon-2(e678)X*.

To make the transgenic nematode expressing the *gst-4::nls::rfp* fusion gene under the control of *gst-4* promoter, we first replaced the CDS (coding sequence) of *gfp* in pPD95.69 (kindly provided by A. Fire, Stanford University) with that of *rfp* in dsRed Monomer (Takara Bio Inc., Japan) to obtain an *nls::rfp* plasmid. Two amplicons, *Pgst-4::gst-4* and *nls::rfp* were obtained using PCR with the primers GST4For_SalI, 5′-GGG TCG ACT TTT GCA GAC TAA AAA TAA CTA CTC TG-3′ and GST4_nls_Rev, 5′-TCC CGG GGA TCC AAC AAT ACT ATC CTT TCT TGT TGC-3′ from the *nls::rfp* plasmid, and primers NLS_rfp_For, 5′-GAT AGT ATT GTT GGA TCC CCG GGA TTG GCC AAA GG-3′ and pPD3UInsRev, 5′-CGT CAT CAC CGA AAC GCG CGA GAC-3′ from the *Pgst-4::gst-4::gfp* construct described above, respectively. Since these two amplicons have a 12 bp overlapping sequence (3′ end of *Pgst-4::gst-4* and 5′ end of *nls::rfp*), a fused single amplicon was produced using PCR with primers GST4For_SalI and pPD3UInsRev from a mixture of the two amplicons (Boulin et al., 2006). A fused single amplicon, 3327 bp long, of the *Pgst-4::gst-4::nls::rfp* fusion gene was purified with agarose gel electrophoresis and concentrated to 100 µg/ml. *Pgst-4::gst-4::nls::rfp* fusion gene was injected, with an equal concentration of pDP#MM016B into the gonadal arms of *unc-119(ed3)* adult hermaphrodites to obtain transgenics having transgenes as an extrachromosomal array. After integration of extrachromosomal arrays into the chromosomes (Mitani, 1995), animals were outcrossed three times with N2 to obtain KHA117 *{chuIs117[unc-119*(+)*, Pgst-4::gst-4::nls::rfp]I}*. We mapped the integrated transgene to LG I as described above.

To make the transgenic nematode expressing the *ctl-1::gfp* fusion gene under the control of *Pctl-1* promoter, an extrachromosomal array of the transgenic animal KHA149 *{unc-119(ed3)III; chuEx149[Pctl-1::Bxy-ctl-1::gfp, pDP#MM016B]}* (Vicente et al., 2015) was integrated into chromosomes as described by Mitani (1995). The transgene-integrated animal was outcrossed twice with N2 to obtain KHA166 *{chuIs166[unc-119(+), Pctl-1::Bxy-ctl-1::gfp]}.*

To make the transgenic nematode expressing the *sod-1::gfp* fusion gene under the control of *Psod-1* promoter, we performed Cas9-mediated *gfp* fluorescence knock ins with a self-excising selection cassette (Dickinson et al., 2015). We accessed the CRISPR design tool of Zhang Lab (http://crispr.mit.edu) and obtained a target site candidate list. To integrate the selected 20 bp sequence into the sgRNA sequence region in the pDD162 vector, the following primers were synthesized, sgRNA_sod1C_kinFor 5′-ACG CGA TTC AGG TAG TCA CTG TTT TAG AGC TAG AAA TAG CAA GT-3′ and sgRNA_Rev 5′**-**CAA GAC ATC TCG CAA TAG G-3′, and ligated into the Cas9 & sgRNA vector pDD162 (Addgene) using a Q5 Site-Directed Mutagenesis Kit (NEB, Japan). Obtained ligated plasmid pKHA1603 (Cas9&sgRNA_sod-1) was confirmed by sequence with a primer sgRNA_ConfirmRev 5′**-**GGT GTG AAA TAC CGC ACA GA-3′.

Two amplicons, sod1C_upstream and sod1C_downstream were obtained using PCR with the primers sod1C_kin_upstFor 5′-AGT CGC CGG CAC TAG ATG TCG AAC CGT GCT GTC GCT G-3′ and sod1C_kin_upstRev 5′-CTC CCG ATG CTC CgT AGc cCT GtG GAG CAG CGA GAG CAA TG-3′ (for sod1C_upstream, 598 bp), and sod1C_kin_dwnstFor 5′-ATG ACA AGA GAC TAG aaT GAc tac ctg aat cgc gtc tct g-3′ and sod1C_kin_dwnstRev 5′-TTA TCG ATT TCC TAG gag att gtc cgt acg ctt tgc gtc-3′ (for sod1C_downstream, 709 bp) from the genomic DNA of the *C. elegans* N2. Two amplicons were ligated with the FP-SEC vector pDD282 (Addgene) to obtain the homologous repair plasmid pKHA1605 (sod-1::GFP^SEC^3×Flag). Two plasmids were mixed (50 ng/μl of pKHA1603 and 10 ng/μl of pKHA1605) and injected into the gonad arm of the *C. elegans* N2 to obtain KHA168 {*sod-1::[chuSi169 gfp::SEC::3×flag]II}*. After heat shock treatment and outcrossing with N2, we finally obtained KHA169 {*sod-1::[chuSi169 gfp::3×flag]II}*.

### Nematicidal assay

All treatment assays were carried out at 20°C. *C. elegans* young adults or dauer larvae were washed with M9 buffer and adjusted to about 20 nematodes per 50 μl. Nematodes were put into each well of a 96-well plate with each concentration of α-terthienyl (Tokyo Chemical Industry Co., Ltd, Japan) and incubated for 24, 48 and 72 h. For PAT treatment, 96-well plates were exposed to UV irradiation (UV-A, 32.5 cm distance) for 30 min (Nivsarkar et al., 1991; Huang et al., 2017) before incubation. Final concentrations of α-terthienyl used were 0, 1, 2.5 and 5 μM (in M9 buffer with 2% DMSO) when photoactivated, or 0, 5, 10 and 25 μM (in M9 buffer with 2% DMSO) without photoactivation. The maximum concentration of α-terthienyl was set to 25 μM since at higher concentrations the compound started to precipitate in the M9 buffer with 2% DMSO. Katiki et al. (2011) and our preliminary experiment (data not shown) indicated that *C. elegans* have tolerance up to 2% DMSO. Nematodes were considered dead if no movements were observed after mechanical stimulation.

*M. incognita* J2s were washed with sterilized water and adjusted to about 20 nematodes per 50 μl. Nematodes were put into each well of a 96-well plate with each concentration of α-terthienyl (in sterilized water with 2% DMSO) with or without photoactivation (UV-A 30 min), and incubated for 24 h. Nematode mortality was checked as described above.

More than four wells of a 96-well plate were prepared for each concentration (total >80 nematodes per sample) in one experiment, and that experiment was repeated three times independently. Many of these mortality data were not normally distributed (data not shown). The significance of the nematicidal activity was statistically analyzed using Kruskal–Wallis or Mann–Whitney *U*-tests followed by Bonferroni corrections with a software EZR (Saitama Medical Center, Jichi Medical University, Saitama, Japan) (Kanda, 2013).

### Expression patterns of detoxification enzymes

To see the expression patterns of detoxification enzymes in *C. elegans* against α-terthienyl, synchronized transgenics at the young adult stage were obtained and treated with 10 µM of α-terthienyl without photoactivation, and 7.03 mM of acrylamide as a positive control (Hasegawa et al., 2008) for 24 h in the 96-well plates, as described above. Treated nematodes were anaesthetized with 1-phenoxy-2-propanol (1% in M9 buffer), then transferred onto the agar pad (Shaham, 2006), covered with a cover slip, and the gene expression patterns were observed with an LSM710 confocal laser scanning microscope (Zeiss).

### Quantitative RT-PCR

To see the expression changes of the three genes, *gst-4*, *sod-1* and *ctl-1* against α-terthienyl, synchronized *C. elegans* N2 at the young adult stage were obtained and treated with 10 µM of α-terthienyl without photoactivation for 24 h in the 9 cm glass dishes as described above with some modifications. Concentrations of all chemicals and nematode were same as previously, but final volume of each treatment was ×100. After treatment, nematodes were collected and washed with M9 buffer, then immediately frozen in liquid nitrogen, and stored in −80°C freezer. Total RNA was extracted by the RNeasy^®^ Plus Micro Kit (Qiagen, Venlo, Netherlands) following the manufacturer's protocol. Total RNA (adjusted for concentration of 50 ng/μl) was reverse transcribed using random hexamer and PrimeScript RT reagent Kit (Takara Bio). Quantitative RT-PCR was performed following the method Sato et al. (2014). The housekeeping *snb-1* gene was used as an internal control gene for calculation of relative expression levels of each gene. Primers for qRT-PCR were listed in Table S2. Experiments were performed four times using independent nematode samples.

### RNAi silencing

Gene fragments of *C. elegans skn-1* and *wdr-23* were prepared by PCR amplification of *C. elegans* N2 genomic DNA and cloned into the RNAi vector pPD129.36 (kindly provided by A. Fire, Stanford University). The PCR fragment-ligated plasmid or the blank vector pPD129.36 was used to transform *E. coli* HT115 (Kamath et al., 2001). Primers for RNAi constructs are listed in Table S1. For RNAi experiments, synchronized L1 stage nematodes were cultured until they reached the young adult stage for 48 h at 20°C on NGM (containing 50 µg/ml ampicillin and 12.5 µg/ml tetracycline) plates seeded with *E. coli* HT115 transformed with each RNAi plasmid. Synchronized young adult nematodes were then collected and transferred onto the α-terthienyl assay plate and we observed their survival or gene expression, as described above.

## Supplementary Material

Supplementary information

## References

[BIO038646C1] An J. H. and Blackwell T. K. (2003). SKN-1 links *C. elegans* mesendodermal specification to a conserved oxidative stress response. *Gene Dev.* 17, 1882-1893. 10.1101/gad.1107803 12869585 PMC196237

[BIO038646C2] Boulin T., Etchberger J. F. and Hobert O. (2006). Reporter gene fusions. *WormBook*, ed. The *C. elegans* Research Community, *WormBook* 10.1895/wormbook.1.106.1 PMC4781452 18050449

[BIO038646C3] Brenner S. (1974). The genetics of *Caenorhabditis elegans*. *Genetics* 77, 71-94. 4366476 10.1093/genetics/77.1.71 PMC1213120

[BIO038646C4] Cassada R. C. and Russell R. L. (1975). The Dauerlarva, a post-embryonic developmental variant of the nematode *Caenorhabditis elegans*. *Dev. Biol.* 46, 326-342. 10.1016/0012-1606(75)90109-8 1183723

[BIO038646C5] Choe K. P., Przybysz A. J. and Strange K. (2009). The WD40 repeat protein WDR-23 functions with the CUL4/DDB1 ubiquitin ligase to regulate nuclear abundance and activity of SKN-1 in *Caenorhabditis elegans*. *Mol. Cell. Biol.* 29, 2704-2715. 10.1128/MCB.01811-08 19273594 PMC2682033

[BIO038646C6] Dickinson D. J., Pani A. M., Heppert J. K., Higgins C. D. and Goldstein B. (2015). Streamlined genome engineering with a self-excising drug selection cassette. *Genetics* 200, 1035-1049. 10.1534/genetics.115.178335 26044593 PMC4574250

[BIO038646C7] Doonan R., McElwee J. J., Matthijssens F., Walker G. A., Houthoofd K., Back P., Matscheski A., Vanfleteren J. R. and Gems D. (2008). Against the oxidative damage theory of aging: superoxide dismutases protect against oxidative stress but have little or no effect on life span in *Caenorhabditis elegans*. *Gene Dev.* 22, 3236-3241. 10.1101/gad.504808 19056880 PMC2600764

[BIO038646C8] Erkut C. and Kurzchalia T. V. (2015). The *C. elegans* dauer larva as a paradigm to study metabolic suppression and desiccation tolerance. *Planta* 242, 389-396. 10.1007/s00425-015-2300-x 25868548

[BIO038646C9] Evans T. C. (2006). Transformation and microinjection, *WormBook*, ed. The *C. elegans* Research Community, WormBook 10.1895/wormbook.1.108.1

[BIO038646C10] Faizi S., Fayyaz S., Bano S., Iqbal E. Y., Lubna, Siddiqi H. and Naz A. (2011). Isolation of nematicidal compounds from *Tagetes patula* L. Yellow flowers: structure-activity relationship studies against cyst nematode *Heterodera zeae* infective stage larvae. *J. Agric. Food Chem.* 59, 9080-9093. 10.1021/jf201611b 21780738

[BIO038646C11] Gillet F.-X., Bournaud C., Antonino de Souza Júnior J. D. and Grossi-de-Sa M. F. (2017). Plant-parasitic nematodes: towards understanding molecular players in stress responses. *Ann Bot.* 119, 775-789. 10.1093/aob/mcw260 28087659 PMC5378187

[BIO038646C12] Hasegawa K., Miwa S., Isomura K., Tsutsumiuchi K., Taniguchi H. and Miwa J. (2008). Acrylamide-Responsive Genes in the Nematode *Caenorhabditis elegans*. *Toxicol. Sci.* 101, 215-225. 10.1093/toxsci/kfm276 17989133

[BIO038646C13] Henkle-Dührsen K. and Kampkötter A. (2001). Antioxidant enzyme families in parasitic nematodes. *Mol. Biochem. Parasitol.* 114, 129-142. 10.1016/S0166-6851(01)00252-3 11378193

[BIO038646C14] Hooks C. R. R., Wang K.-H., Ploeg A. and McSorley R. (2010). Using marigold (*Tagetes* spp.) as a cover crop to protect crops from plant-parasitic nematodes. *Appl. Soil Ecol.* 46, 307-320. 10.1016/j.apsoil.2010.09.005

[BIO038646C15] Huang Q., Yun X., Rao W. and Xiao C. (2017). Antioxidative cellular response of lepidopteran ovarian cells to photoactivated alpha-terthienyl. *Pestic. Biochem. Physiol.* 137, 1-7. 10.1016/j.pestbp.2016.09.006 28364798

[BIO038646C16] Jones L. M., De Giorgi C. and Urwin P. E. (2011). *C. elegans* as a Resource for Studies on Plant Parasitic Nematodes. In *Genomics and Molecular Genetics of Plant–Nematode Interactions* (ed. Jones J., Gheysen G. and Fenoll C.), pp. 175-220. Switzerland: Springer Nature.

[BIO038646C17] Jordan D. L., Barnes J. S., Corbett T., Bogle C. R., Johnson P. D., Shew B. B., Koenning S. R., Ye W. and Brandenburg R. L. (2008). Crop response to rotation and tillage in Peanut-based cropping systems. *Agron. J.* 100, 1580-1586. 10.2134/agronj2008.0075

[BIO038646C18] Kamath R. S., Martinez-Campos M., Zipperlen P., Fraser A. G. and Ahringer J. (2001). Effectiveness of specific RNA-mediated interference through ingested double-stranded RNA in *Caenorhabditis elegans*. *Genome Biol.* 2, 1-10. 10.1186/gb-2000-2-1-research0002 PMC17598 11178279

[BIO038646C19] Kanda Y. (2013). Investigation of the freely available easy-to-use software ‘EZR’ for medical statistics. *Bone Marrow. Transplant.* 48, 452-458. 10.1038/bmt.2012.244 23208313 PMC3590441

[BIO038646C20] Karssen G. and Moens M. (2006). Root-knot nematodes. In *Plant Nematology* (ed. Perry R. N. and Moens M.), p. 59 Oxford, UK: CABI.

[BIO038646C21] Katiki L. M., Ferreira J. F. S., Zajac A. M., Masler C., Lindsay D. S., Chagas A. C. S. and Amarante A. F. T. (2011). *Caenorhabditis elegans* as a model to screen plant extracts and compounds as natural anthelmintics for veterinary use. *Vet. Parasitol.* 182, 264-268. 10.1016/j.vetpar.2011.05.020 21680095

[BIO038646C22] Kushida A. and Kondo N. (2015). A simple method for the detection and discrimination of Pratylenchus and Meloidogyne species in nematode communities. *Nematol. Res.* 45, 101-114. 10.3725/jjn.45.101

[BIO038646C23] Lindblom T. H. and Dodd A. K. (2006). Xenobiotic detoxification in the nematode *Caenorhabditis elegans*. *J. Exp. Zool.* 305, 720-730. 10.1002/jez.a.324 PMC2656347 16902959

[BIO038646C24] Mitani S. (1995). Genetic regulation of mec-3 gene expression implicated in the specification of the mechanosensory neuron cell types in *Caenorhabditis elegans*. *Dev. Growth Differ.* 37, 551-557. 10.1046/j.1440-169X.1995.t01-4-00010.x 37281420

[BIO038646C25] Nguyen T., Sherrett P. J., Huang H.-C., Yang C. S. and Pickett C. B. (2003). Increased protein stability as a mechanism that enhances Nrf2-mediated transcriptional activation of the antioxidant response element. *J. Biol. Chem.* 278, 4536-4541. 10.1074/jbc.M207293200 12446695

[BIO038646C26] Nishiyama H., Ngan B. T., Nakagami S., Ejima C., Ishida T. and Sawa S. (2015). Protocol for root-knot nematode culture by a hydroponic system and nematode inoculation to *Arabidopsis*. *Nematol. Res.* 45, 45-49. 10.3725/jjn.45.45

[BIO038646C27] Nivsarkar M., Kumar G. P., Laloraya M. and Laloraya M. M. (1991). Superoxide dismutase in the anal gills of the mosquito larvae of *Aedes aegypti*: its inhibition by alpha-terthienyl. *Arch. Insect Biochem. Physiol.* 16, 249-255. 10.1002/arch.940160404 1799676

[BIO038646C28] Nivsarkar M., Cherian B. and Padh H. (2001). Alpha-terthienyl: a plant-derived new generation insecticide. *Curr. Sci.* 81, 667-672.

[BIO038646C29] Ntalli N. G. and Caboni P. (2012). Botanical nematicides: a review. *J. Agric. Food Chem.* 60, 9929-9940. 10.1021/jf303107j 22973877

[BIO038646C30] Oka Y., Koltai H., Bar-Eyal M., Mor M., Sharon E., Chet I. and Spiegel Y. (2000). New strategies for the control of plant-parasitic nematodes. *Pest Manag. Sci.* 56, 983-988. 10.1002/1526-4998(200011)56:11<983::AID-PS233>3.0.CO;2-X

[BIO038646C31] Park S.-K., Tedesco P. M. and Johnson T. E. (2009). Oxidative stress and longevity in *Caenorhabditis elegans* as mediated by SKN-1. *Aging Cell* 8, 258-269. 10.1111/j.1474-9726.2009.00473.x 19627265 PMC2762118

[BIO038646C33] Porta-de-la-Riva M., Fontrodona L., Villanueva A. and Cerón J. (2012). Basic *Caenorhabditis elegans* methods: synchronization and observation. *J. Vis. Exp.* 64, e4019 10.3791/4019 PMC3607348 22710399

[BIO038646C34] Sato K., Yoshiga T. and Hasegawa K. (2014). Activated and inactivated immune responses in *Caenorhabditis elegans* against *Photorhabdus luminescens* TT01. *SpringerPlus* 3, 274 10.1186/2193-1801-3-274 25279274 PMC4171960

[BIO038646C35] Shaham S. (2006). Methods in Cell Biology, *WormBook*, ed. The *C. elegans* Research Community, WormBookm 10.1895/wormbook.1.49.1

[BIO038646C36] Steiner G. (1941). Nematodes parasitic on and associated with roots of marigolds (*Tagetes* hybrids). *Proc. Biol. Soc. Wash.* 54, 31-34.

[BIO038646C37] Stiernagle T. (2006). Maintenance of *C. elegans*, *WormBook*, ed. The *C. elegans* Research Community, WormBook 10.1895/wormbook.1.101.1

[BIO038646C38] Tang L. and Choe K. P. (2015). Characterization of *skn-*1/*wdr-*23 phenotypes in *Caenorhabditis elegans*; pleiotrophy, aging, glutathione, and interactions with other longevity pathways. *Mech. Ageing Dev.* 149, 88-98. 10.1016/j.mad.2015.06.001 26056713

[BIO038646C39] Tawe W. N., Eschbach M.-L. and Walter R. D. and Henkle-Dührsen K. (1998). Identification of stress-responsive genes in *Caenorhabditis elegans* using RT-PCR differential display. *Nucleic Acids Res.* 26, 1621-1627. 10.1093/nar/26.7.1621 9512531 PMC147444

[BIO038646C40] Vanfleteren J. R. and De Vreese A. (1995). The gerontogenes *age-*1 and *daf-*2 determine metabolic rate potential in aging *Caenorhabditis elegans*. *FASEB J.* 9, 1355-1361. 10.1096/fasebj.9.13.7557026 7557026

[BIO038646C41] Vicente C. S. L., Ikuyo Y., Shinya R., Mota M. and Hasegawa K. (2015). Catalases induction in high virulence pinewood nematode *Bursaphelenchus xylophilus* under hydrogen peroxide-induced stress. *PLoS ONE* 10, e0123839 10.1371/journal.pone.0123839 25894519 PMC4404050

[BIO038646C42] Wang K. H., Hooks C. R. and Ploeg A. (2007). *Protecting Crops from Nematode Pests: Using Marigold as an Alternative to Chemical Nematicides*. *Plant Disease Publication PD-*35, pp. 1-6. Manoa, Hawaii, University of Hawaii at Manoa, College of Tropical Agriculture and Human Resources.

[BIO038646C43] Zechmeister L. and Sease J. W. (1947). A blue-fluorescing compound, terthienyl, isolated from marigolds. *J. Am. chem. Soc.* 69, 273-275. 10.1021/ja01194a032 20292435

[BIO038646C44] Zeng Q., Huang H., Zhu J., Fang Z., Sun Q. and Bao S. (2013). A new nematicidal compound produced by *Streptomyces albogriseolus* HA10002. *Antonie Leeuwenhoek* 103, 1107-1111. 10.1007/s10482-013-9890-8 23444037 PMC3621998

