# Supplementary material for: Nematicidal actions of the marigold exudate α-terthienyl: oxidative stress-inducing compound penetrates nematode hypodermis
Source: Biol Open. 2019 Mar 29;8(4):bio038646. doi: 10.1242/bio.038646 (PMC6504006; doi:10.1242/bio.038646)
Supplement: Supplementary information [file biolopen-8-038646-s1.pdf]

Supplementary materials

Table S1. Primers for RNAi

Ceskn1EcoRI_For, 5' – GGAATTCGGCCAATCCAAATATGATTATCCA – 3'
Ceskn1EcoRI_Rev, 5' – GGAATTCGGGCAGCAACCTTGTTCTTTCCG – 3'
Cewdr-23_For, 5' – AGGGAACAACATATTGCATTTAGT – 3'
Cewdr-23_RevEcoRI, 5' – GGGAATTCTTGGGATGATCGTATGGTGCAA – 3'

Table S2. Primers for quantitative RT-PCR

Cegst-4_qPCRFor, 5' – TGCTCAATGTGCCTTACGAGGA – 3'
Cegst-4_qPCRRev, 5' – GGGAAGCTGGCCAAATGGAG – 3'
Cesod-1_qPCRFor, 5' – GAAGCTGGAGCCGATGGAGT – 3'
Cesod-1_qPCRRev, 5' – GGCCAACGACAGTGTTTGGA – 3'
Cectl-1_qPCRFor, 5' –AGCCACGTCAGTTCTGGGAG– 3'
Cectl-1_qPCRRev, 5' – TCCTCCAAACAGCCACCCAA – 3'
Cesnb-1_qPCRFor, 5' – TGGAGCGTGATCAGAAGTTGTC – 3'
Cesnb-1_qPCRRev, 5' – TCCACCAATACTTGCGCTTCAG – 3'

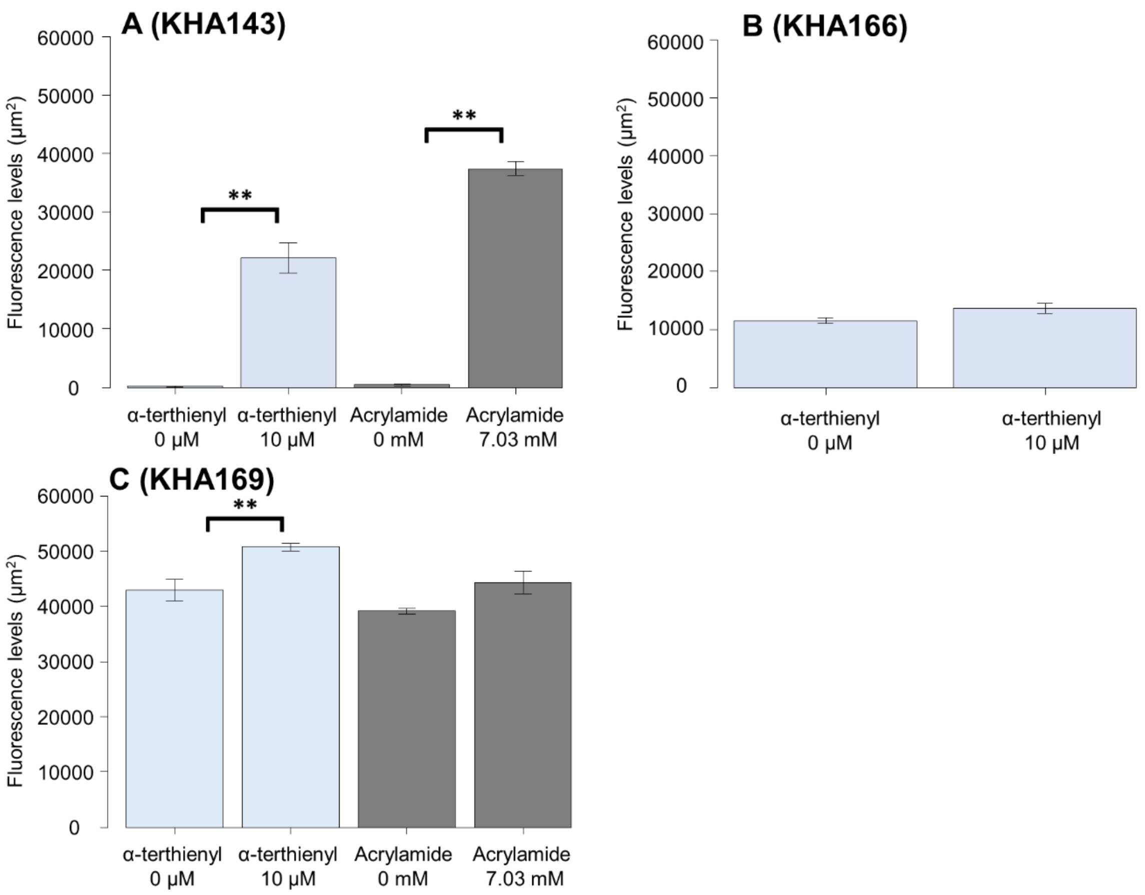

Figure S1. Quantitative analysis of GST-4, CTL-1, and SDO-1 expressions, treated with chemicals. (A) GST-4 expressions in the transgenic animal KHA143 were significantly induced when treated with 10 μM of α-terthienyl and 7.03 mM of acrylamide for 24 h. (B) CTL-1 expressions in the transgenic animal KHA166 were not induced when treated with 10 μM of α-terthienyl. (C) SOD-1 expressions in the transgenic animal KHA169 were significantly induced when treated with 10 μM of α-terthienyl. Induction of SOD-1 was not significantly but such trait was seen when treated with 7.03 mM of acrylamide for 24 h. GFP fluorescence signal densities from the individual nematodes (pixels/μm^2) were measured from the photomicrographs with the Image J (NIH, https://imagej.nih.gov/ij/). Average ± S.E. of each values was calculated from >8 individuals from three (α-terthienyl) or two (acrylamide) independent experiments (***P* < 0.005, Mann-Whitney U test).

Biology Open • Supplementary information

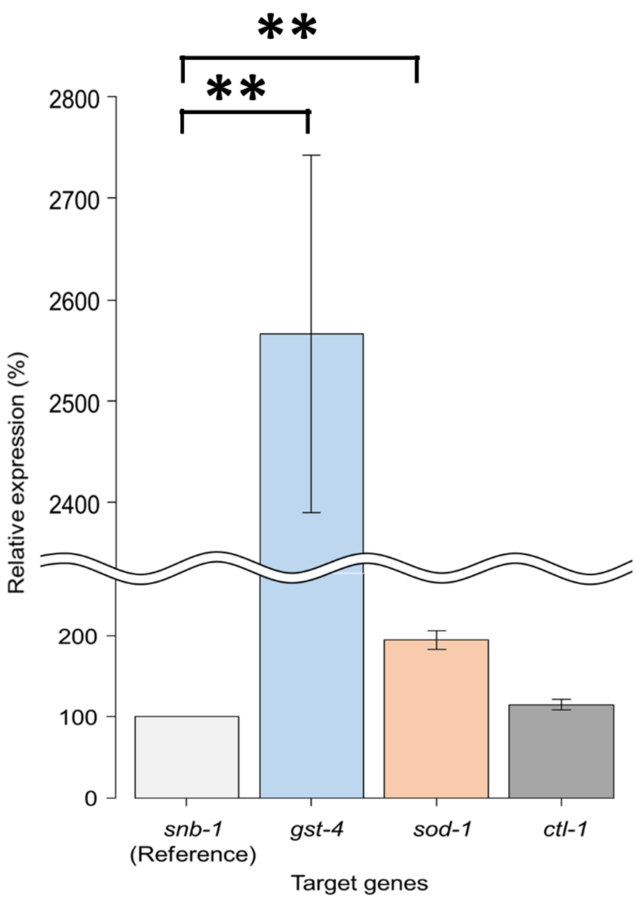

Figure S2. Quantitative RT-PCR analysis of *gst-4*, *sod-1*, and *ctl-1*expression changes after treated with 10 µM of α-terthienyl for 24 h. Expression changes of *gst-4*, *sod-1*, and *ctl-1* in *C. elegans* N2 were 2,567 ± 169 %, 195 ± 11.3%, and 115 ± 6.55 % when treated with 10 µM of α-terthienyl. The housekeeping *snb-1* gene was used as an internal control gene for calculation of relative expression levels of each gene. Values are means ± SE of from four independent experiments （**P < 0.005, Mann-Whitney U test followed by Bonferroni correction ）.

Biology Open • Supplementary information

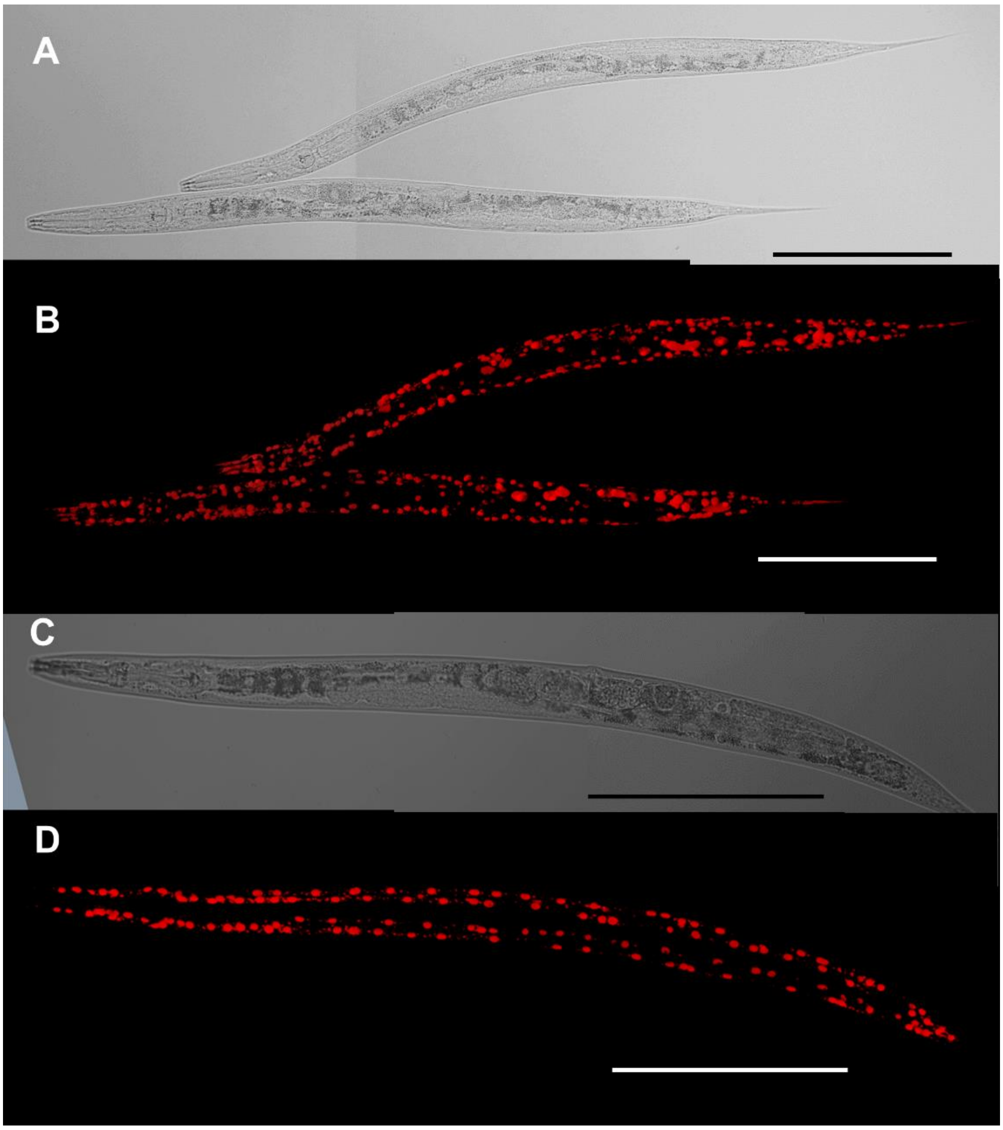

Figure S3. GST-4::NLS::RFP expression patterns in the transgenic nematode KHA117 *{chuIs117[unc-119*(+), *Pgst-4::gst-4::nls::rfp]I}*. (A, B) GST-4::NLS::RFP expression was observed in almost all somatic nuclei when treated with 7.03 mM of acrylamide for 24 h. (C, D) GST-4::NLS::RFP expression was clearly observed in hypodermal cell nuclei when treated with 10 μM of α-terthienyl for 24 h. Scale bars, 200 μm.

Biology Open • Supplementary information

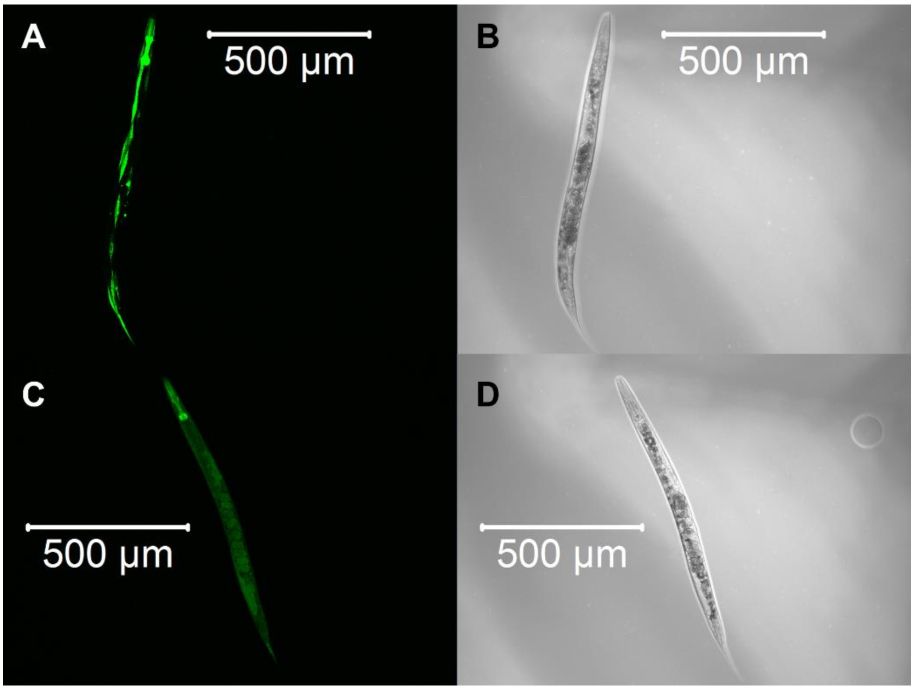

Figure S4. Knock down of *skn-1* by RNAi suppresses the inductions of GST-4::GFP and SOD-1::GFP expression even after treatment with 7.03 mM of acrylamide except for muscles. (A, B) GST-4::GFP in KHA143 *{chuIs143[unc-119(+), Pgst-4::gst-4::gfp]II}* was observed only in pharynx and body-wall muscle after treatment with 7.03 mM of acrylamide for 24 h. (C, D) Induction of SOD-1::GFP expression in KHA169 *{sod-1::gfp [chuSi169 gfp::3×flag]II}* was observed only in pharynx after treatment with 7.03 mM of acrylamide for 24 h. Scale bars, 500 µm.

Biology Open • Supplementary information
